# Comparison of pulse pressure variation with radial arterial systolic, diastolic and pulse transit time interval variation in pediatric patients undergoing liver transplantation

**DOI:** 10.1186/cc12141

**Published:** 2013-03-19

**Authors:** YJ Moon, IG Jun, WJ Shin, BH Sang, GS Hwang

**Affiliations:** 1 Asan Medical Center, Seoul, South Korea

## Introduction

In pediatric patients, dynamic preload indices to predict fluid responsiveness have conflicting results in comparison with adults. A recent study demonstrated that pulse pressure variation (PPV) ≥16% has provided an accurate method for predicting fluid responsiveness in pediatric congenital heart surgery. We aimed to compare PPV and respiratory systolic, diastolic and pulse transit time interval variation (STV, DTV and PTTV, respectively) as predictors of fluid responsiveness during pediatric liver transplantation.

## Methods

A total of 61 data from 16 pediatric patients, median age 5.4 years (range 0.1 to 9 years), were retrospectively evaluated from electrically recorded radial arterial and central venous pressure (CVP) waveform. The time from the onset of systolic upstroke to the dicrotic notch was defined as the systolic time interval (STI), and the time from dicrotic notch to the beginning of systolic upstroke was defined as the diastolic time interval (DTI). The time from peak R wave on electrocardiography to the onset of systolic upstroke was defined as the pulse transit time (PTT) interval. STV was calculated by averaging of three consecutive respiratory cycles with the following: (STI_maximum _- STI_minimum_) / STI_mean_. The same method was used for calculating DTV, PTTV and PPV. STV, DTV and PTTV were corrected by cardiac period. Averaged CVP was used as a static preload index. PPV threshold ≥16% was used to discriminate fluid responsiveness. Receiver operating characteristic (ROC) curves and Pearson's correlation analysis were used for the comparison.

## Results

PPV showed correlations with STV, DTV and PTTV (*r *= 0.65, 0.57 and 0.60, respectively), but less with CVP (*r *= -0.30). Area under ROC curves (AUC) of STV, DTV, PTTV and CVP were 0.834, 0.872, 0.832 and 0.613, respectively. Cutoff values of STV, DTV, PTTV and CVP were 7.7% (sensitivity/specificity, 0.80/0.83), 7.7% (sensitivity/specificity, 0.70/0.88), 8.7% (sensitivity/specificity 0.67/1.0) and 3.1% (sensitivity/ specificity 0.50/0.85), respectively.

## Conclusion

This study shows that STV, DTV and PTTV can be used as a surrogate for PPV ≥16%, suggesting that this novel method can be used to predict hemodynamic response during pediatric surgery.
